# Star-shaped and linear π-conjugated oligomers consisting of a tetrathienoanthracene core and multiple diketopyrrolopyrrole arms for organic solar cells

**DOI:** 10.3762/bjoc.12.142

**Published:** 2016-07-14

**Authors:** Hideaki Komiyama, Chihaya Adachi, Takuma Yasuda

**Affiliations:** 1 International Institute for Carbon Neutral Energy Research (WPI-I2CNER), Kyushu University, 744 Motooka, Nishi-ku, Fukuoka 819-0395, Japan; 2 INAMORI Frontier Research Center (IFRC), Kyushu University; 3 Center for Organic Photonics and Electronics Research (OPERA), Kyushu University

**Keywords:** bulk heterojunction, diketopyrrolopyrrole, organic solar cells, star-shaped oligomers, tetrathienoanthracene

## Abstract

Solution-processable star-shaped and linear π-conjugated oligomers consisting of an electron-donating tetrathienoanthracene (TTA) core and electron-accepting diketopyrrolopyrrole (DPP) arms, namely, TTA-DPP4 and TTA-DPP2, were designed and synthesized. Based on density functional theory calculations, the star-shaped TTA-DPP4 has a larger oscillator strength than the linear TTA-DPP2, and consequently, better photoabsorption property over a wide range of visible wavelengths. The photovoltaic properties of organic solar cells based on TTA-DPP4 and TTA-DPP2 with a fullerene derivative were evaluated by varying the thickness of the bulk heterojunction active layer. As a result of the enhanced visible absorption properties of the star-shaped π-conjugated structure, better photovoltaic performances were obtained with relatively thin active layers (40–60 nm).

## Introduction

Solution-processable organic semiconductors have been intensively studied as key materials for low-cost, flexible, and large-area optoelectronic devices, including organic solar cells (OSCs) [1–4]. OSCs have drawn much attention as promising next-generation renewable energy sources because abundant sun-light energy can be directly converted into electricity. Recently, the power conversion efficiencies (PCEs) of OSCs based on small molecules with bulk heterojunction (BHJ) architectures have rapidly increased to approximately 10% [5], approaching those of state-of-the-art polymer-based OSCs [6–7]. To produce high-performance OSCs, the donor materials are required to possess suitable frontier orbital energy levels, high carrier mobility, excellent film-forming ability, and good miscibility with a fullerene derivative as an acceptor. Moreover, strong visible light photoabsorption ability for visible light is vital for the donor materials.

**Figure 1:**
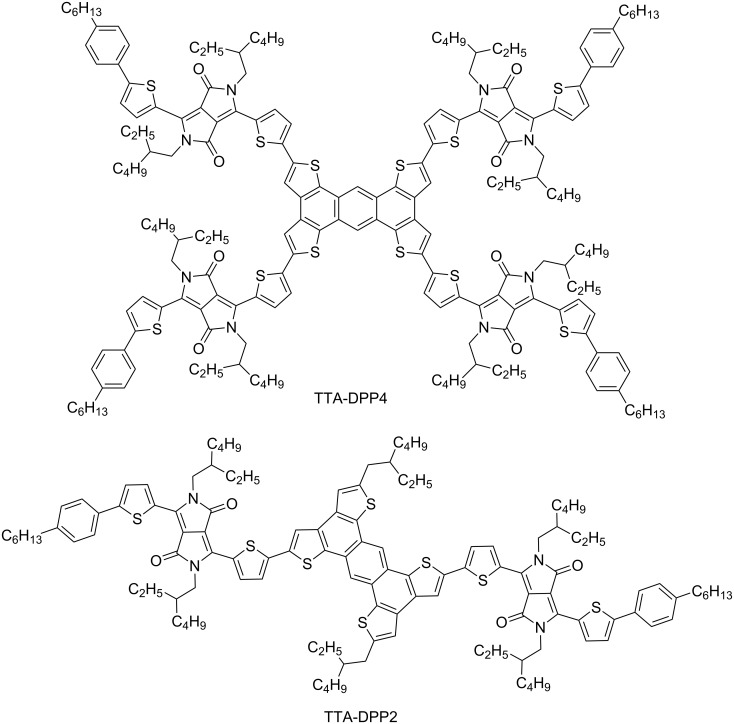
Chemical structures of TTA-DPP4 and TTA-DPP2.

Star-shaped molecules tethering multiple π-conjugated arms are capable of harvesting incident light effectively owing to their extended dimensionality, in comparison to their linear analogues. Star-shaped π-conjugated molecules consisting of hexa-*peri*-hexabenzocoronene [8–14], pyrene [15–17], and triphenylamine [18–23] central cores have been developed as donor materials for BHJ-OSCs. The design of star-shaped π-conjugated molecules offers possibilities for not only further enhancement of the photoabsorption ability but also for the improvement of molecular packing, solubility, and film-forming ability. However, the impact of star-shaped molecular structures on optoelectronic functionality has not been fully characterized because of the small variation in the central core units researched so far. Herein, we report the design and synthesis of a new star-shaped π-conjugated oligomer composed of an electron-donating tetrathienoanthracene (TTA) core coupled with multiple electron-accepting diketopyrrolopyrrole (DPP) arms, and its linear analogue, TTA-DPP4 and TTA-DPP2 (Figure 1). TTA can be regarded as a promising central core unit for star-shaped π-conjugated oligomers, and has previously been utilized as a building block of semiconducting polymers for OSCs [24] and organic field-effect transistors [25–27]. To the best of our knowledge, however, there has been no precedent on TTA-based star-shaped and linear π-conjugated oligomers that can be applied to OSCs.

## Results and Discussion

### Molecular design and synthesis

**Figure 2:**
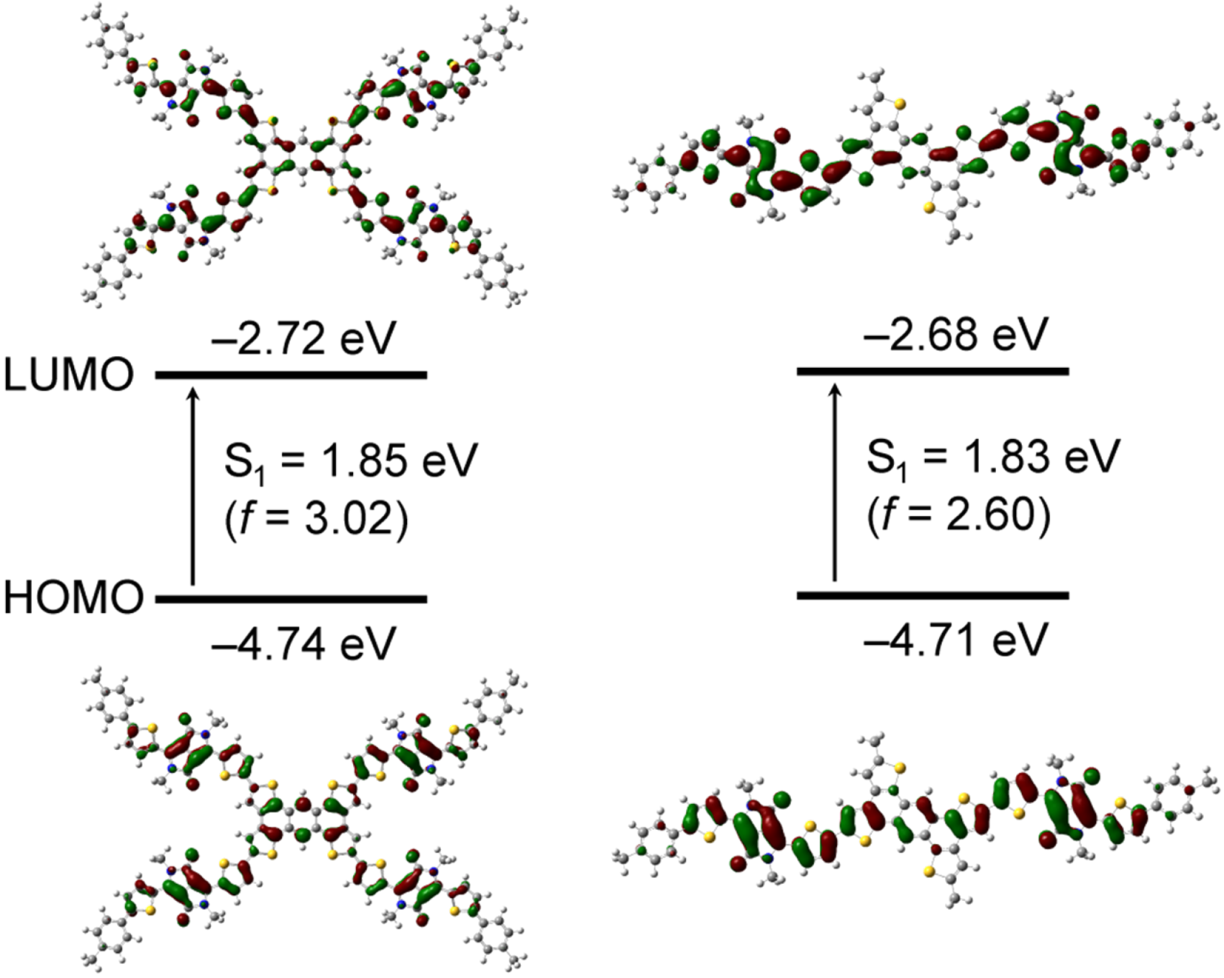
HOMO and LUMO distributions, calculated energy levels, and associated oscillator strengths (*f*) for TTA-DPP4 and TTA-DPP2 calculated at the B3LYP/6-31G(d,p) level. The arrows indicate the first singlet–excited state (S_1_) transition. The alkyl chains are modified to methyl groups to simplify the calculations.

To develop star-shaped and linear π-conjugated oligomers with acceptor–donor–acceptor electronic structures, an electron-donating TTA unit was coupled with electron-accepting diketopyrrolopyrrole (DPP)-based chromophoric units, which are well-known building blocks used in OSCs [28–31]. Such molecular structures facilitate intramolecular charge transfer, rendering the molecules with narrower bandgaps, and broad and strong light absorptions. To gain insight into the geometric and electronic structures of TTA-DPP4 and TTA-DPP2, density functional theory (DFT) calculations were performed at the B3LYP/6-31G(d,p) level. The calculated energy levels and the respective frontier orbital distributions for these molecules are displayed in Figure 2. The HOMOs and LUMOs of TTA-DPP4 and TTA-DPP2 are well-expanded across the entire π-conjugated structure. Both molecules possess similar HOMO and LUMO energy levels of approximately −4.7 and −2.7 eV, respectively. The star-shaped structure has an apparent effect on its oscillator strength (*f*) such that a larger *f* value leads to a larger absorption coefficient. Therefore, the four-armed TTA-DPP4 (*f* = 3.02) is expected to show better photoabsorption ability than the two-armed TTA-DPP2 (*f* = 2.60) because of the two-dimensionally extended π-conjugated structure.

**Scheme 1:**
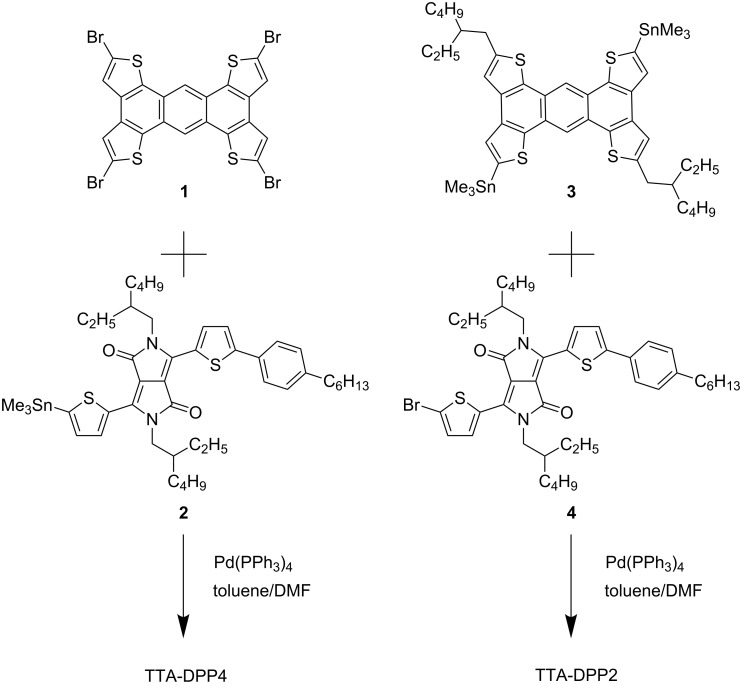
Synthesis of TTA-DPP4 and TTA-DPP2.

**Figure 3:**
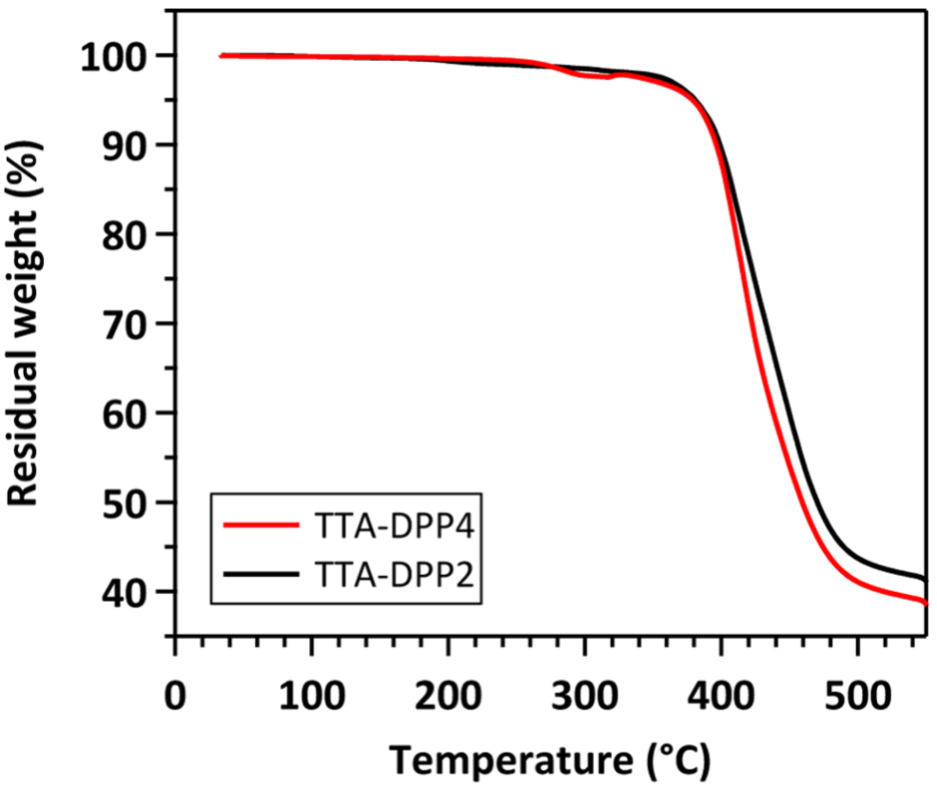
TGA curves of TTA-DPP4 and TTA-DPP2 at a heating rate of 10 °C min^-1^ under N_2_.

The synthetic routes to TTA-DPP4 and TTA-DPP2 are outlined in Scheme 1 (see also Experimental section and Supporting Information File 1 for details). TTA-DPP4 and TTA-DPP2 were synthesized in 44% and 49% yields, respectively, via the palladium-catalyzed Stille cross-coupling reaction. Both TTA-DPP4 and TTA-DPP2 were soluble in common organic solvents, such as chloroform, toluene, and chlorobenzene. The thermal stability of these compounds was analyzed by thermogravimetric analysis (TGA). As shown in Figure 3, the 5% weight-loss temperature of TTA-DPP4 and TTA-DPP2, under N_2_ atmosphere, was determined to be 378 and 380 °C, respectively, suggesting the high thermal stability for these compounds as donor materials.

### Optical properties

**Figure 4:**
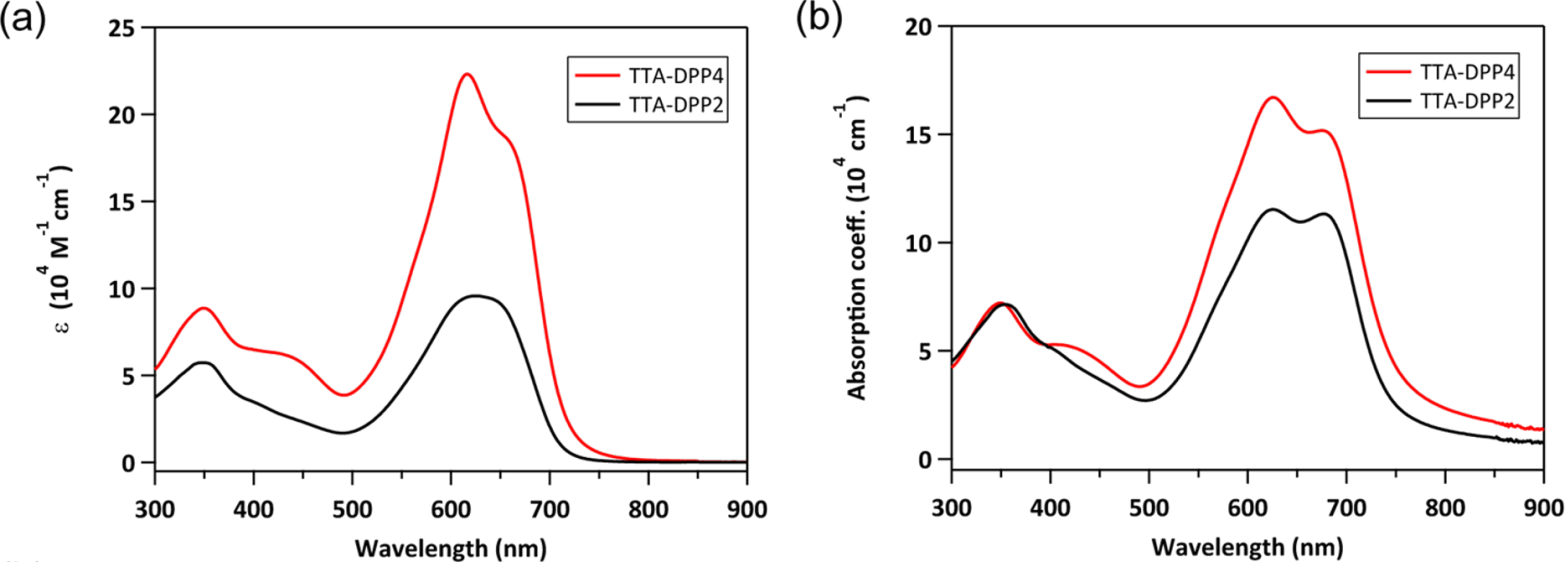
UV–vis absorption spectra of TTA-DPP4 (red) and TTA-DPP2 (black) in (a) chloroform solutions and (b) thin films.

The UV–vis absorption spectra of TTA-DPP4 and TTA-DPP2 in chloroform solutions and as-spun thin films are depicted in Figure 4, and the corresponding photophysical data are summarized in Table 1. In chloroform solution, TTA-DPP4 and TTA-DPP2 exhibit strong and broad absorptions with two characteristic bands: a shorter-wavelength absorption (300–400 nm) arising from π–π* transitions and a longer-wavelength absorption (500–700 nm) originating from intramolecular charge transfer (ICT) transitions between the electron-donating TTA and the electron-accepting DPP units. Evidently, the maximum absorption coefficient (ε) of TTA-DPP4 (22.3 × 10^4^ M^−1^ cm^−1^) is more than twice that of TTA-DPP2 (9.6 × 10^4^ M^−1^ cm^−1^), which is consistent with the increment in the oscillator strength revealed by the DFT calculation results. The ICT absorption peaks are slightly red-shifted and broadened in the solid thin films as compared to those in the chloroform solutions, which can be ascribed to molecular aggregation in the condensed solid state. The optical band gaps of TTA-DPP4 and TTA-DPP2 are estimated to be 1.64 and 1.65 eV, respectively, from the onset positions of their absorption bands. The absorption coefficient (α) of TTA-DPP4 (16.7 × 10^4^ cm^−1^) in the thin film is still considerably larger than that of TTA-DPP2 (11.5 × 10^4^ cm^−1^), indicating the enhanced photoabsorption property of the four-armed TTA-DPP4 in the visible region. The HOMO energy levels of TTA-DPP4 and TTA-DPP2 in the thin films were determined to be –5.36 and –5.40 eV, respectively, via photoelectron yield spectroscopy (Figure S1 in Supporting Information File 1). The LUMO energy levels were calculated to be –3.72 and –3.75 eV for TTA-DPP4 and TTA-DPP2, respectively. Because of their deep-lying HOMO levels and sufficient LUMO offsets (>0.5 eV), both TTA-DPP4 and TTA-DPP2 can serve as electron-donor materials in combination with [6,6]-phenyl-C_71_-butyric acid methyl ester (PC_71_BM) as an acceptor materials, which has a LUMO level of –4.3 eV.

**Table 1:** Photophysical data of TTA-based molecules.

	Solution^a^		Thin film^b^		HOMO^c^ [eV]	LUMO^d^ [eV]	*E*_g_^e^ [eV]
	λ_max_ [nm]	ε [10^4^ M^−1^ cm^−1^]	λ_max_ [nm]	α [10^4^ cm^−1^]			

TTA-DPP4	616	22.3	625, 676	16.7	−5.36	−3.72	1.64
TTA-DPP2	623	9.6	626, 677	11.5	−5.40	−3.75	1.65

^a^Measured in chloroform solutions (10^−5^ M); ^b^thin films spin-coated from chloroform solution onto quartz substrate; ^c^determined by photoelectron yield spectroscopy in spin-coated thin films; ^d^determined from the HOMO and optical energy gap (*E*_g_): LUMO = HOMO + *E*_g_; ^e^derived from the absorption onset of the thin films.

### Photovoltaic properties

The photovoltaic properties of TTA-DPP4 and TTA-DPP2 were also evaluated. BHJ-OSCs were fabricated using TTA-DPP4 and TTA-DPP2 as electron-donor materials and PC_71_BM as an electron-acceptor material, with an inverted device configuration of glass/ITO/ZnO/donor:PC_71_BM(1:1.5, w/w)/MoO_3_/Ag. A 30 nm thick ZnO layer was deposited on the ITO-coated glass via the sol–gel method (see Experimental section). The active layer was then spin-coated from a solution of the donor material and PC_71_BM in a mixed solvent of chloroform/1,8-diiodooctane (DIO) (98:2, v/v). The thickness of the active layer was controlled within the range of ca. 40–90 nm by varying the rotation speed during spin-coating. A 6 nm thick MoO_3_ layer as a hole extraction layer and a 100 nm thick Ag anode were vacuum-deposited on the active layer. The photovoltaic properties of the fabricated BHJ-OSCs were evaluated under simulated AM 1.5G illumination at an intensity of 100 mW cm^−2^ (1 sun).

**Figure 5:**
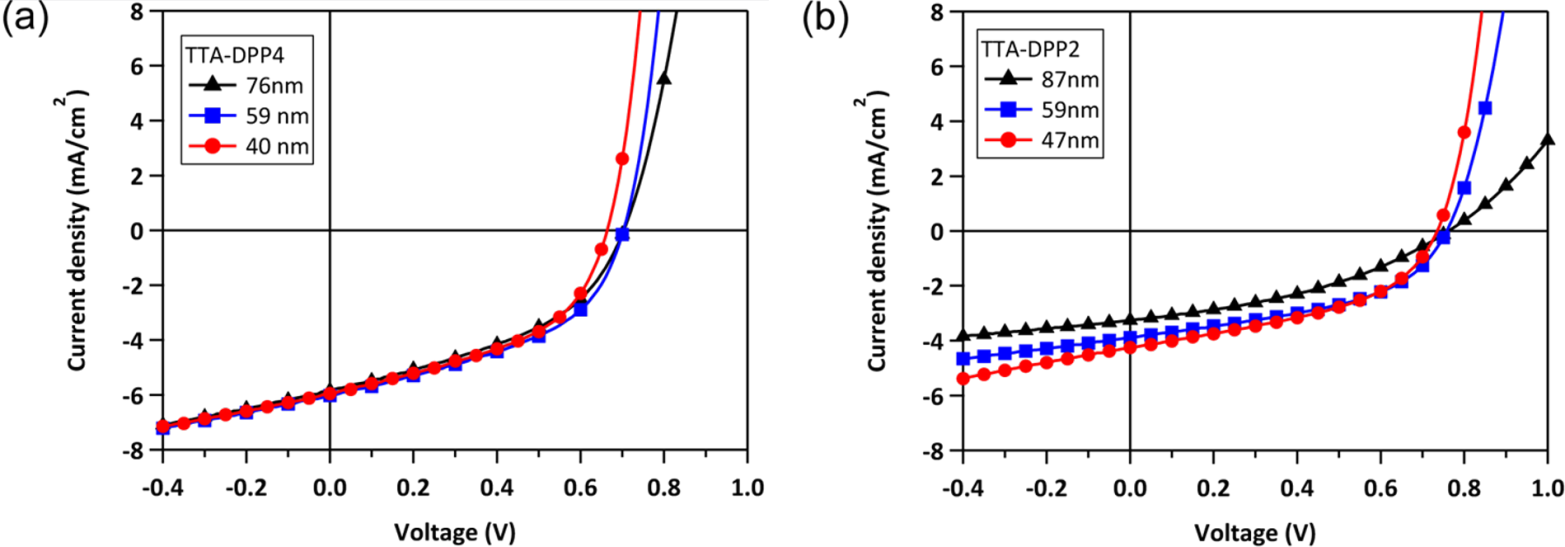
*J*–*V* characteristics of BHJ-OSCs based on (a) TTA-DPP4:PC_71_BM (1:1.5, w/w) and (b) TTA-DPP2:PC_71_BM (1:1.5, w/w) with different active layer thicknesses, measured under simulated AM 1.5G, 100 mW cm^−1^ illumination.

**Figure 6:**
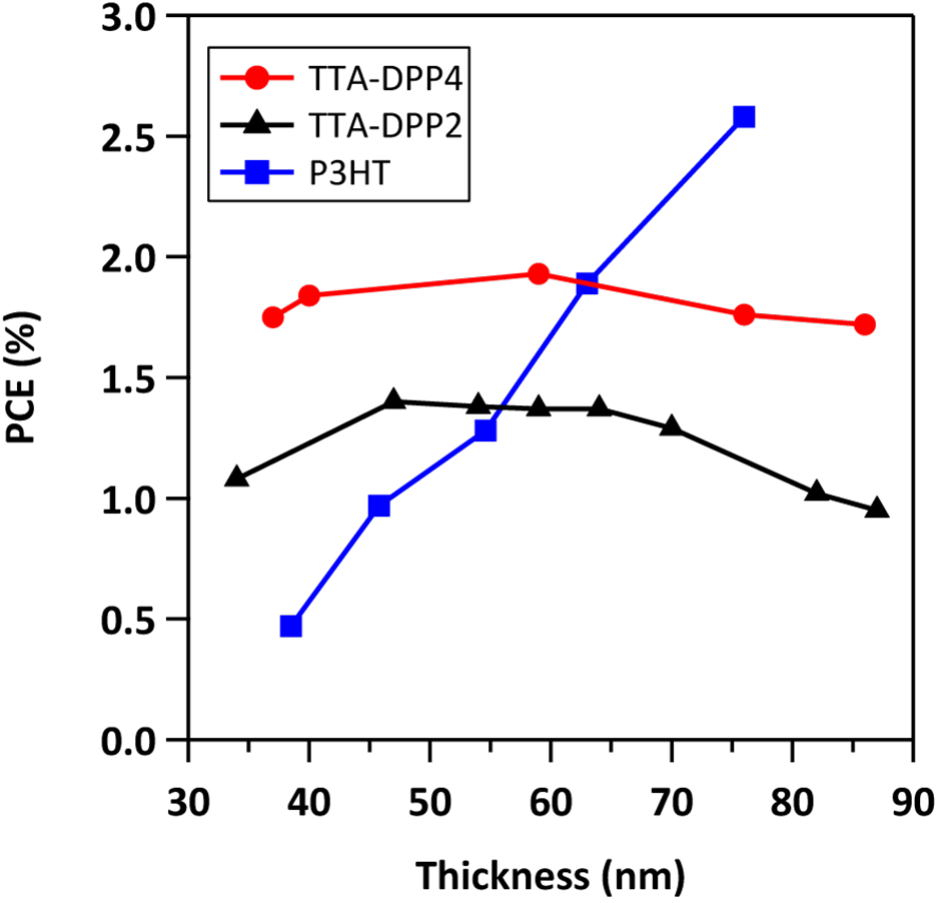
Relationship between active layer thickness and power conversion efficiency (PCE) for TTA-DPP4, TTA-DPP2, and P3HT-based BHJ-OSCs (see also Figures S2–S4 in the Supporting Information File 1 for detailed photovoltaic data).

Figure 5 shows the current density–voltage (*J*–*V*) characteristics of the BHJ-OSCs based on TTA-DPP4 and TTA-DPP2 with different active layer thicknesses. The dependence of the active layer thickness on PCE is presented in Figure 6, and the representative photovoltaic parameters are listed in Table 2. For the TTA-DPP4-based devices, the photovoltaic properties were not influenced much when changing the thickness of the active layer in the range of ca. 40–90 nm, providing PCEs of 1.7–2.0%. Apparently, the TTA-DPP4-based OSCs showed better photovoltaic performance with a thinner active layer (40–80 nm), as compared to conventional poly(3-hexylthiophene) (P3HT)-based devices. A similar effect of active layer thickness on PCE was also observed in TTA-DPP2-based devices, where a PCE of 1.4% was obtained with an active layer thickness of 47 nm. In contrast, as presented in Figure 6 and Table 2, the PCEs of P3HT-based devices steeply decreased with decreasing active layer thickness in the range of 40–80 nm, agreeing with the previously reported results [32]. For most of the donor materials, including polymers and small molecules reported, the best photovoltaic performances were generally achieved with an active layer thickness of around 100 nm. However, TTA-DPP4 and TTA-DPP2 presented the highest PCEs with much thinner active layers of approximately 40–60 nm. Moreover, the PCEs of TTA-DPP4-based devices were higher than those of TTA-DPP2, presumably because of its larger absorption coefficient resulting from its star-shaped molecular structure with a two-dimensionally expanded π-conjugated backbone.

**Table 2:** Photovoltaic parameters for inverted BHJ-OSCs based on TTA-DPP4 or TTA-DPP2:PC_71_BM and P3HT:PC_61_BM under simulated AM 1.5 G, 100 mW cm^−1^ illumination.

Donor	Thickness [nm]	*J*_SC_ [mA/cm^2^]	*V*_OC_ [V]	FF [%]	PCE [%]

TTA-DPP4	76 ± 3	5.84	0.70	42.9	1.76
TTA-DPP4	59 ± 1	6.02	0.70	45.6	1.93
TTA-DPP4	40 ± 2	5.95	0.66	46.7	1.84

TTA-DPP2	87 ± 1	3.26	0.76	44.9	0.95
TTA-DPP2	59 ± 1	3.89	0.76	46.4	1.37
TTA-DPP2	47 ± 2	4.25	0.74	44.7	1.40

P3HT	76 ± 1	7.32	0.56	62.8	2.58
P3HT	55 ± 1	5.60	0.43	52.4	1.28
P3HT	39 ± 1	2.97	0.32	50.0	0.47

## Conclusion

In this study, the first attempt to introduce a TTA unit as central electron-donating core into star-shaped and linear π-conjugated oligomers was demonstrated. Multiple electron-accepting DPP arms were attached to the electron-donating TTA core to form star-shaped TTA-DPP4 and linear TTA-DPP2 that have acceptor–donor–acceptor electronic structures. TTA-DPP4 showed a better photoabsorption property than TTA-DPP2 because of its larger oscillator strength, as expected from the DFT calculations. The BHJ-OSCs based on TTA-DPP4 and TTA-DPP2 showed good photovoltaic properties even with thin active layers of 40–60 nm. This behavior was highly different from that of the reported general polymer- and small-molecule-based OSCs. A star-shaped molecular structure containing a two-dimensionally extended π-conjugated system is a promising electronic system for designing photovoltaic organic materials, as a result of its excellent photoabsorption properties.

## Experimental

### General methods

Matrix-assisted laser desorption ionization time-of-flight (MALDI–TOF) mass spectra were collected on a Bruker Daltonics Autoflex III spectrometer using dithranol as the matrix. Elemental analysis was carried out using a YANACO CHN coder MT-6. Thermogravimetric analysis (TGA) was performed using a Hitachi High-Tech Science TG/DTA7300 with a heating rate of 10 °C min^−1^ under N_2_ atmosphere. UV–vis absorption spectra were recorded on a JASCO V-670Y spectrometer. Photoelectron yield spectra were recorded on a Riken-Keiki AC-2 ultraviolet photoelectron spectrometer. The thickness of photoactive layers was measured using a Bruker DektakXT system.

### Synthesis

All reactions were carried out under N_2_ atmosphere using standard Schlenk techniques. All starting materials, unless otherwise specified, were purchased from commercial suppliers and used without further purification. 2,5,9,12-Tetrabromoanthra[1,2-*b*:4,3-*b*':5,6-*b*'':8,7-*b*''']tetrathiophene (**1**) [25], 2,5-bis(2-ethylhexyl)-3-(5-(4-hexylphenyl)thiophen-2-yl)-6-(5-(trimethylstannyl)thiophen-2-yl)-2,5-dihydropyrrolo[3,4-*c*]pyrrole-1,4-dione (**2**) [30,33], (5,12-bis(2-ethylhexyl)anthra[1,2-*b*:4,3-*b*':5,6-*b*'':8,7-*b*''']tetrathiophene-2,9-diyl)bis(trimethylstannane) (**3**) [24,27], and 3-(5-bromothiophen-2-yl)-2,5-bis(2-ethylhexyl)-6-(5-(4-hexylphenyl)thiophen-2-yl)-2,5-dihydropyrrolo[3,4-*c*]pyrrole-1,4-dione (**4**) [30] were synthesized according to the reported procedures. Detailed synthetic schemes for these compounds are provided in the Supporting Information File 1.

**Synthesis of TTA-DPP4:** To a mixture of **1** (0.19 g, 0.27 mmol) and **2** (2.29 g, 2.70 mmol) in a mixture of dry DMF (10 mL) and dry toluene (20 mL) was added Pd(PPh_3_)_4_ (0.016 g, 0.014 mmol). The mixture was stirred for 38 h at 120 °C. After cooling to room temperature, the reaction mixture was poured into water and then extracted with chloroform. The combined organic layers were washed with water and dried over anhydrous Na_2_SO_4_. After filtration and evaporation, the product was purified by silica gel column chromatography (eluent: chloroform/hexane 4:1, v/v) to provide TTA-DPP4 as a dark purple solid. This compound was further purified by recycling preparative gel permeation chromatography (GPC; eluent: chloroform) prior to use (yield = 0.36 g, 44%). MS (MALDI–TOF) *m*/*z*: [*M*]^+^ calcd for 3133.42; found 3133.51; anal. calcd (%) for C_190_H_226_N_8_O_8_S_12_: C, 72.80; H, 7.27; N, 3.57; found: C, 71.29; H, 7.15; N, 3.53. Well-resolved NMR signals could not be obtained for both TTA-DPP4 and TTA-DPP2 in CDCl_3_ or DMSO-*d*_6_ even at elevated temperatures due to the macromolecular nature of the compounds (Figure S5 in Supporting Information File 1). The experimental results of isotope pattern deconvolution in mass spectra of TTA-DPP4 and TTA-DPP2 showed good agreements with the theoretical isotope patterns, supporting their chemical structures.

**Synthesis of TTA-DPP2:** This compound was prepared in a similar fashion to TTA-DPP4, using **3** (0.31 g, 0.33 mmol) and **4** (0.53 g, 0.69 mmol), and Pd(PPh_3_)_4_ (0.015 g, 0.013 mmol). The product was purified by silica gel column chromatography (eluent: chloroform/hexane 1:1, v/v) and GPC to give TTA-DPP2 as a dark purple solid (yield = 0.32 g, 49%). MS (MALDI–TOF) *m*/*z*: [*M*]^+^ calcd for 1991.95; found 1991.67; anal. calcd (%) for C_122_H_150_N_4_O_4_S_8_: C, 73.52; H, 7.59; N, 2.81; found: C, 73.33; H, 7.53; N, 2.84.

### Fabrication and evaluation of organic solar cells

The OSC devices were fabricated and tested by using similar procedures described in reference [30]. Pre-patterned ITO-coated glass substrates were cleansed sequentially by sonicating in detergent solution, deionized water, acetone, and isopropanol for 15 min each, and then subjected to UV/ozone treatment for 30 min. A thin layer (~30 nm) of ZnO was prepared by spin-coating (at 5000 rpm) a precursor solution of zinc acetate (0.50 g) and ethanolamine (0.14 g) in 2-methoxyethanol (5 mL), followed by baking at 200 °C for 10 min under air. The photoactive layer was then prepared by spin-coating from a chloroform solution containing the donor material and PC_71_BM, after passing through a 0.45 μm PTFE membrane filter. Finally, a 6 nm thick MoO_3_ layer and a 100 nm-thick Ag layer were thermally evaporated on top of the active layer under high vacuum through a shadow mask, defining an active area of 0.04 cm^2^ for each device. Current density–voltage (*J*–*V*) measurements for the fabricated OSCs were conducted on a computer-controlled Keithley 2400 source measure unit in air, under simulated AM 1.5G solar illumination at 100 mW cm^−2^ (1 sun), using a Xe lamp-based Bunko-Keiki SRO-25 GD solar simulator. The light intensity was calibrated using a standard silicon photovoltaic reference cell.

## Supporting Information

File 1 Synthesis of compounds **1**–**13**, evaluation of HOMO levels for TTA-DPP4 and TTA-DPP2, photovoltaic properties of TTA-DPP4, TTA-DPP2, and P3HT-based OSCs and ^1^H NMR spectra of TTA-DPP4 and TTA-DPP2.
