# Supplementary material for: Star-shaped and linear π-conjugated oligomers consisting of a tetrathienoanthracene core and multiple diketopyrrolopyrrole arms for organic solar cells
Source: Beilstein J Org Chem. 2016 Jul 14;12:1459–66. doi: 10.3762/bjoc.12.142 (PMC4979874; doi:10.3762/bjoc.12.142)
Supplement: File 1 — Synthesis of compounds 1–13, evaluation of HOMO levels for TTA-DPP4 and TTA-DPP2, photovoltaic properties of TTA-DPP4, TTA-DPP2, and P3HT-based OSCs and 1H NMR spectra of TTA-DPP4 and TTA-DPP2. [file Beilstein_J_Org_Chem-12-1459-s001.pdf]

Supporting Information

for

Star-shaped and linear π-conjugated oligomers consisting of a tetrathienoanthracene core and multiple diketopyrrolopyrrole arms for organic solar cells

Hideaki Komiyama*[,1,2], Chihaya Adachi[1,3] and Takuma Yasuda*[,2]

Address: [1]International Institute for Carbon Neutral Energy Research (WPI-I2CNER), Kyushu University, 744 Motooka, Nishi-ku, Fukuoka 819-0395, Japan, [2]INAMORI Frontier Research Center (IFRC), Kyushu University and [3]Center for Organic Photonics and Electronics Research (OPERA), Kyushu University.

Email: Hideaki Komiyama - komiyama@ifrc.kyushu-u.ac.jp; Takuma Yasuda - yasuda@ifrc.kyushu-u.ac.jp

*Corresponding author

Synthesis of compounds 1–13, evaluation of HOMO levels for TTA-DPP4 and TTA-DPP2, photovoltaic properties of TTA-DPP4, TTA-DPP2, and P3HT-based OSCs and ^{1}H NMR spectra of TTA-DPP4 and TTA-DPP2

Table of Contents:

Synthesis and characterization of compounds 1–13

Figure S1: Photoelectron yield spectra of TTA-DPP4 andTTA-DPP2

Figure S2: Photovoltaic properties of TTA-DPP4-based OSCs

Figure S3: Photovoltaic properties of TTA-DPP2-based OSCs

Figure S4: Photovoltaic properties of P3HT-based OSCs

Figure S5: ^{1}H NMR spectra of TTA-DPP4 and TTA-DPP2

Synthesis and characterization of compounds 1–13.

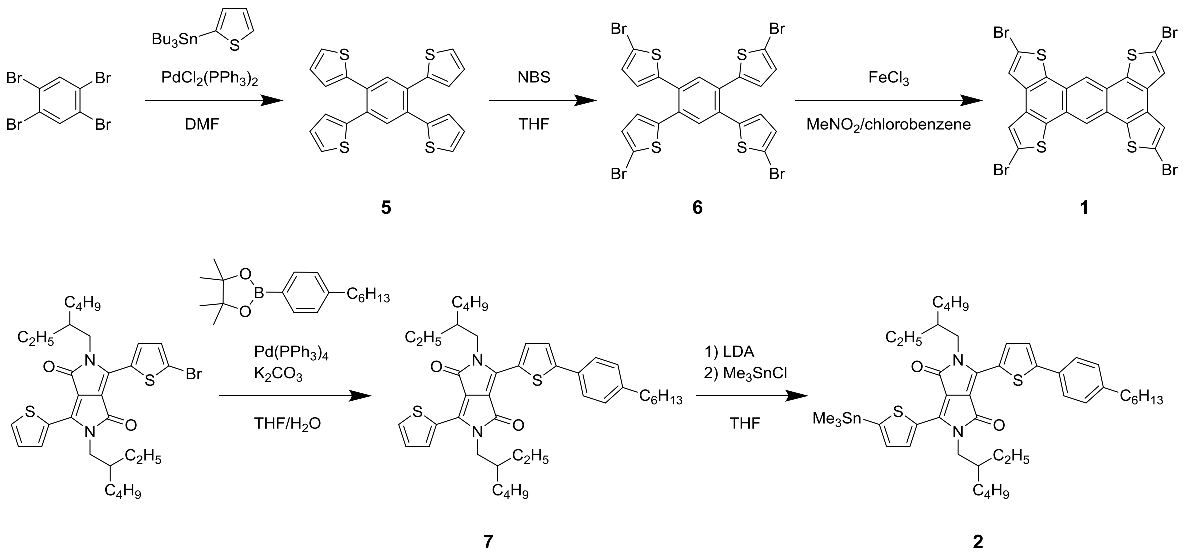

Scheme S1: Synthetic routes for compounds **1** and **2**.

2-[2,4,5-Tris(2-thienyl)phenyl]thiophene (5).

To a mixture of 1,2,4,5-tetrabromobenzene (10.0 g, 25.3 mmol) and 2-(tributylstannyl)thiophene (40 mL, 129 mmol) in dry DMF (5 mL) were added $PdCl_2$ (321 mg, 1.80 mmol) and triphenylphosphine (949 mg, 3.62 mmol). The mixture was stirred for 16 h at 130 °C. After cooling to room temperature, the formed precipitate was collected by filtration and washed with hexane to afford **5** as a white solid (9.00 g, 87%). ^{1}H NMR (400 MHz, $CDCl_3$): δ 7.67 (s, 2H), 7.30 (dd, J = 4.0 Hz, 1.2 Hz, 4H), 6.98 (m, 8H). MS (MALDI-TOF) m/z: $[M]^+$ calcd 406.00; found 405.78.

2-Bromo-5-[2,4,5-tris(5-bromo-2-thienyl)phenyl]thiophene (6).

To a stirred solution of **5** (5.00 g, 12.3 mmol) in dry THF (250 mL) was added slowly *N*-bromosuccinimide (NBS, 13.2 g, 73.8 mmol) at 0 °C. The mixture was allowed to warm to room temperature and stirred overnight in the dark. The resulting white precipitate was filtered and rinsed with water and acetone before drying under vacuum to provide **6** as a white solid (5.07 g, 57 %). ^{1}H NMR (400 MHz, $CDCl_3$): δ 7.50 (s, 2H), 6.97 (d, J = 4.0 Hz, 4H), 6.74 (d, J = 4.0 Hz, 4H). MS (MALDI-TOF) m/z: $[M]^+$ calcd 717.64; found 717.80.

2,5,9,12-Tetrabromoanthra[1,2-*b*:4,3-*b*':5,6-*b*'':8,7-*b*''']tetrathiophene (1).

A solution of $FeCl_3$ (3.39 g, 20.8 mmol) in nitromethane (50 mL) was added dropwise to a solution of **6** (2.53 g, 3.40 mmol) in chlorobenzene (100 mL), and the reaction mixture was stirred for 1 h. The resulting precipitate was collected by filtration, rinsed with dichloromethane, and dried in air. The solid was then washed with HCl aq (10%) and methanol, and dried under vacuum to provide crude product as a yellow-green solid (2.41 g, 96%). This compound was further purified by sublimation before using. MS (MALDI-TOF) *m*/*z*: $[M]^+$ calcd 717.60; found 717.90. Given the low solubility of this compound ^{1}H NMR data could not be obtained.

2,5-Bis(2-ethylhexyl)-3-(5-(4-hexylphenyl)thiophen-2-yl)-6-(thiophen-2-yl)-2,5-dihydropyrrolo[3,4-*c*]pyrrole-1,4-dione (7).

To a stirred solution of 3-(5-bromothiophen-2-yl)-2,5-bis(2-ethylhexyl)-6-(thiophen-2-yl)-2,5-dihydropyrrolo[3,4-*c*]pyrrole-1,4-dione (2.50 g, 4.14 mmol) and 2-(4-hexylphenyl)-4,4,5,5-tetramethyl-[1,3,2]dioxaborolane (1.70 g, 4.55 mmol) in dry THF (40 mL) were added $Pd(PPh_3)_4$ (0.24 g, 0.21 mmol) and aqueous K_2CO_3 (2.0 M, 20 mL). The mixture was stirred for 17 h at 60 °C. After cooling to room temperature, the reaction mixture was poured into water and extracted with chloroform. The combined organic layers were washed with water and dried over anhydrous Na_2SO_4. After filtration and evaporation, the product was purified silica gel column chromatography (eluent: $CHCl_3$), recrystallized from chloroform/methanol, and dried under vacuum to give **7** as a dark violet solid (yield = 2.75 g, 97%). ^{1}H NMR (400 MHz, $CDCl_3$): δ 8.97 (d, J = 4.0 Hz, 1H), 8.86 (dd, J = 4.0 Hz, 1.0 Hz, 1H), 7.61-7.55 (m, 3H), 7.41 (d, J = 4.0 Hz, 1H), 7.27-7.18 (m, 3H), 4.08-3.96 (m, 4H), 2.62 (t, J = 7.6 Hz, 2H), 1.96-1.80 (m, 2H), 1.67-1.60 (m, 2H), 1.41-1.19 (m, 22H), 0.91-0.81 (m, 15H). MS (MALDI-TOF) *m*/*z*: $[M]^+$ calcd 686.38; found 686.36.

2,5-Bis(2-ethylhexyl)-3-(5-(4-hexylphenyl)thiophen-2-yl)-6-(5-(trimethylstannyl)thiophen-2-yl)-2,5-dihydropyrrolo[3,4-*c*]pyrrole-1,4-dione (2).

n-Butyllithium (*n*-BuLi, 2.6 M, 1.8 mL, 4.50 mol) was stirred in dry THF (20 mL) at −10 °C under N_2. Diisopropylamine (0.8 mL, 5.40 mmol) was added to the solution dropwise and the reaction mixture was stirred for 30 min. The solution was warmed up to room temperature to prepare a fresh solution of lithium diisopropylamine (LDA) in THF. To a solution of **7** (2.06 g, 3.00 mmol) in dry THF (40 mL) was added dropwise the freshly prepared LDA solution at –78 °C. The mixture was stirred for 1 h at that temperature. Then, trimethyltin chloride (0.90 g, 4.50 mmol) was slowly added to the mixture at −78 °C. The reaction mixture was further stirred overnight at room

temperature. The reaction mixture was poured into water followed by extraction with hexane. The combined organic layers were washed with water and dried over anhydrous Na_2SO_4. After filtration and evaporation, the crude product was concentrated under vacuum to give **2** as a sticky dark purple oil (yield = 2.45 g, 98%). This oil was directly used for next step without any further purification.

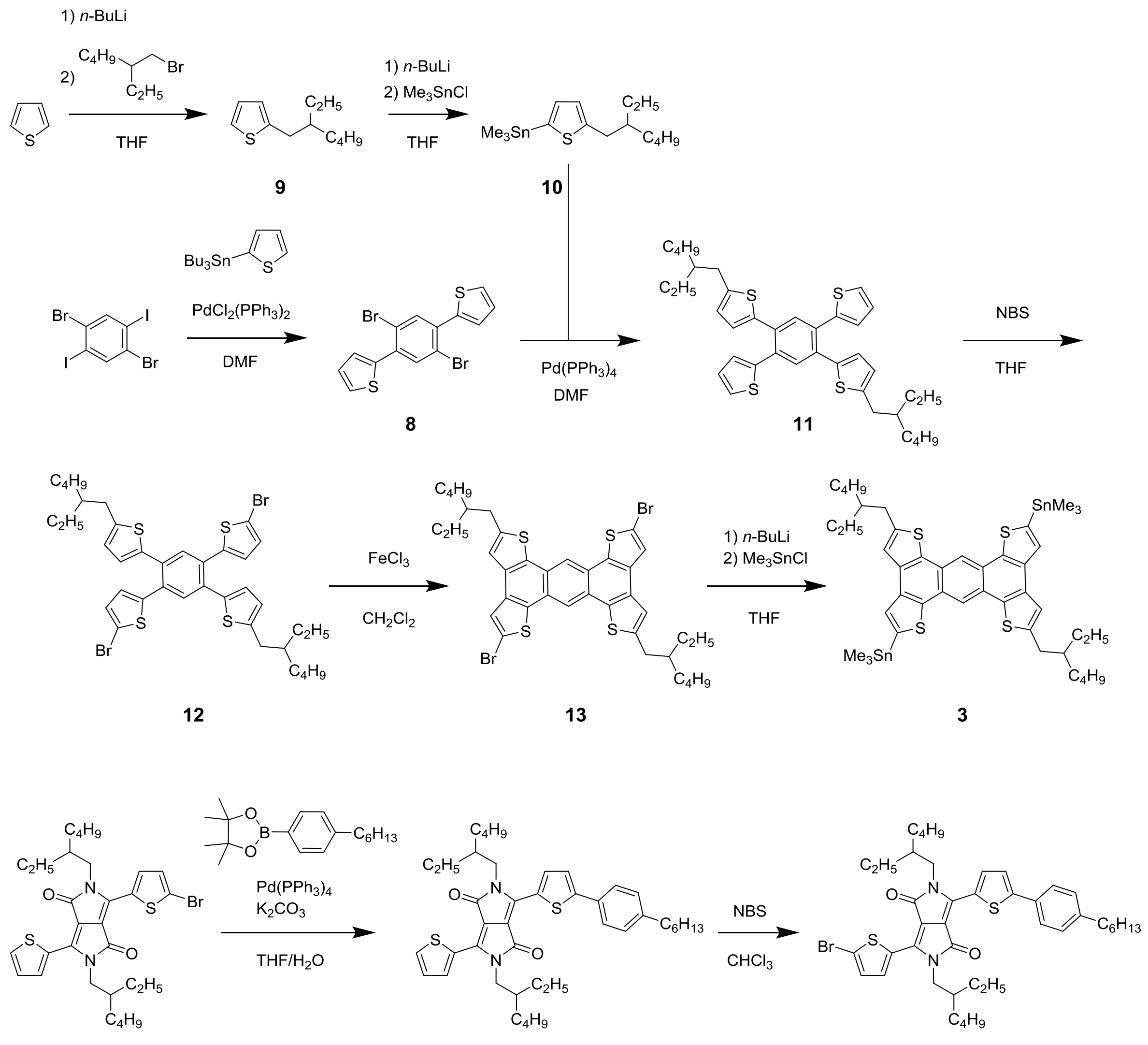

Scheme S2: Synthetic routes for compounds **3** and **4**.

2-(2-Ethylhexyl)thiophene (9).

To a stirred solution of thiophene (9.70 g, 115 mmol) in dry THF (50 mL) was added dropwise *n*-BuLi (2.6 M, 52.1 mL, 138 mmol) at –78 °C. The mixture was stirred for 1 h at that temperature. Then, 3-(bromomethyl)heptane (21.6 g, 121 mmol) was added dropwise to the mixture at –78 °C. The reaction mixture was further stirred overnight at 60 °C. The reaction mixture was poured into water followed by extraction with hexane. The combined organic layers were washed with water, and dried over anhydrous Na_2SO_4. After filtration and evaporation, the crude product was purified by silica gel column chromatography (eluent: hexane) and dried under vacuum to give **9** as a color less oil (yield = 13.2 g, 58%). ^{1}H NMR (400 MHz, $CDCl_3$): δ 7.11 (d, *J* = 3.4 Hz, 1H), 6.91 (m, *J* = 3.4 Hz, 1H), 6.75 (d, *J* = 3.4 Hz, 1H), 2.75 (d, *J* = 6.8 Hz, 2H), 1.58 (m, 1H), 1.32-1.28 (m, 8H), 0.92-0.88 (m,6H).

(5-(2-Ethylhexyl)thiophen-2-yl)trimethylstannane (10).

To a solution of **9** (13.0 g, 66.0 mmol) in dry THF (150 mL) was added dropwise *n*-BuLi (2.6M, 31 mL, 79.2 mmol) at –78 °C. The mixture was stirred for 1 h at that temperature. Then, trimethyltin chloride (17.1 g, 85.8 mmol) was slowly added to the mixture at –78 °C. The reaction mixture was further stirred overnight at room temperature. The resulting mixture was poured into water, and then extracted with hexane. The combined organic layers were washed with water and dried over anhydrous Na_2SO_4. After filtration and evaporation, the crude product was concentrated under vacuum to provide **10** as a yellow oil (yield = 24.0 g, 100%) which was used without any further purification. ^{1}H NMR (400 MHz, $CDCl_3$): δ 7.00 (d, *J* = 3.2 Hz, 1H), 6.87 (d, *J* = 3.2 Hz, 1H), 2.79 (d, *J* = 6.8 Hz, 2H), 1.58 (m, 1H), 1.36-1.26 (m, 8H), 0.90-0.87 (m,6H), 0.34 (s, 9H).

1,4-Bis(2-thienyl)-2,5-dibromobenzene (8).

To a stirred solution of 2-(tributylstannyl)thiophene (20.7 g, 55.3 mmol) and 1,4-dibromo-2,5-diiodobenzene (13.5 g, 27.7 mmol) in dry DMF (100 mL) was added $Pd(PPh_3)_2Cl_2$ (0.78 g, 1.12 mmol). The mixture was stirred for 40 h at 80 °C. After cooling to room temperature, the reaction mixture was poured into water and then extracted with toluene. The combined organic layers were washed with water and dried over anhydrous Na_2SO_4. After filtration and evaporation, the crude product was recrystallized from methanol to provide **8** as a white solid (yield = 10.3 g, 93%). ^{1}H NMR (400 MHz, $CDCl_3$): δ 7.80 (s, 2H), 7.44 (d, *J* = 5.2 Hz, 2H), 7.36 (d, *J* = 3.6 Hz, 2H), 7.13 (m, *J* = 3.6 Hz, 2H). MS (MALDI-TOF) *m*/*z*: $[M]^+$ calcd 397.84; found 397.63.

5,5'-(2,5-Di(thiophen-2-yl)-1,4-phenylene)bis(2-(2-ethylhexyl)thiophene) (11).

To a stirred solution of **10** (12.6 g, 35.2 mmol) and **8** (6.40 g, 16.0 mmol) in dry DMF (45 mL) was added $Pd(PPh_3)_4$ (0.74 g, 0.64 mmol). The mixture was stirred overnight at 85 °C. After cooling to room temperature, the reaction mixture was poured into water and then extracted with dichloromethane. The combined organic layers were washed with water and dried over anhydrous Na_2SO_4. After filtration and evaporation, the product was purified by silica gel column chromatography (eluent: hexane) and dried under vacuum to afford **11** as a white solid (yield = 8.58 g, 85%). ^{1}H NMR (400 MHz, $CDCl_3$): δ 7.59 (s, 2H), 7.26 (d, *J* = 4.0 Hz, 2H), 6.96-6.93 (m, 4H), 6.71 (d, *J* = 3.5 Hz, 2H), 6.59 (d, *J* = 3.5 Hz, 2H), 2.67 (d, *J* = 6.8 Hz, 4H), 1.53 (m, 2H), 1.32-1.22 (m, 16H), 0.88-0.82 (m, 12H). MS (MALDI-TOF) *m*/*z*: $[M]^+$ calcd 630.25; found 630.29.

5,5'-(2,5-Bis(5-(2-ethylhexyl)thiophen-2-yl)-1,4-phenylene)bis(2-bromothiophene) (12).

To a stirred solution of **11** (7.62 g, 12.1 mmol) in dry THF (100 mL) was added slowly NBS (4.73 g, 26.6 mmol) at 0 °C. The mixture was allowed to warm to room temperature and stirred overnight. The reaction mixture was poured into water and then extracted with dichloromethane. The combined organic layers were washed with water and dried over anhydrous Na_2SO_4. After filtration and evaporation, the product was purified by silica gel column chromatography (eluent: chloroform/hexane 1:1, v/v) and dried under vacuum to afford **12** as a sticky yellow oil (yield = 8.10 g, 85%). ^{1}H NMR (400 MHz, $CDCl_3$): δ 7.54 (s, 2H), 6.91 (d, *J* = 4.0 Hz, 2H), 6.78-6.74 (m, 4H), 6.65 (d, *J* = 3.5 Hz, 2H), 2.73 (d, *J* = 6.8 Hz, 4H), 1.52 (m, 2H), 1.36-1.29 (m, 16H), 0.91-0.88 (m, 12H).

2,9-Dibromo-5,12-bis(2-ethylhexyl)anthra[1,2-*b*:4,3-*b*':5,6-*b*'':8,7-*b*''']tetrathiophene (13).

To a stirred solution of **12** (2.76 g, 3.50 mmol) in dichloromethane (400 mL) was added dropwise a solution of $FeCl_3$ (3.41 g, 21.0 mmol) in dichloromethane (50 mL). After stirring for 30 min, methanol (1000 mL) was added to the mixture and the reaction solution was further stirred for 30 min. The formed precipitate was collected by filtration and rinsed with water, methanol, and hot hexane. The crude product was recrystallization form hexane to afford **13** as a yellow solid (692 mg, 25%). ^{1}H NMR (400 MHz, $CDCl_3$): δ 8.58 (s, 2H), 8.15 (s, 2H), 7.00 (s, 2H), 2.97 (d, *J* = 6.8 Hz, 4H), 1.87 (m, 2H), 1.51-1.44 (m, 16H), 1.02-0.94 (m, 12H). MS (MALDI-TOF) *m*/*z*: $[M]^+$ calcd 782.04; found 782.11.

(5,12-Bis(2-ethylhexyl)anthra[1,2-b:4,3-b':5,6-b'':8,7-b''']tetrathiophene-2,9-diyl)-bis(trimethylstannane) (3).

To a solution of **13** (1.10 g,1.40 mmol) in dry THF (50 mL) was added dropwise *n*-BuLi (2.6 M, 1.35 mL, 3.50 mmol) at –78 °C. The mixture was stirred for 1 h at that temperature. Then, trimethyltin chloride (0.70 g, 3.5 mmol) was slowly added to the mixture at −78 °C for 1 h. The reaction mixture was further stirred overnight at room temperature. The resulting mixture was poured into water, and then extracted with chloroform. The combined organic layers were washed with water and dried over anhydrous Na_2SO_4. After filtration and evaporation, the crude product was recrystallized form isopropanol and hexane to give **3** as a yellow solid (yield = 857 mg, 64%). ^{1}H NMR (400 MHz, $CDCl_3$): δ 8.84 (s, 2H), 7.78 (s, 2H), 7.45 (s, 2H), 2.98 (d, *J* = 6.8 Hz, 4H), 1.58 (m, 2H), 1.41-1.37 (m, 16H), 0.97-0.92 (m, 12H), 0.52 (m, 18H). MS (MALDI-TOF) *m*/*z*: $[M]^+$ calcd 954.15; found 954.32.

3-(5-Bromothiophen-2-yl)-2,5-bis(2-ethylhexyl)-6-(5-(4-hexylphenyl)thiophen-2-yl)-2,5-dihydropyrrolo[3,4-*c*]pyrrole-1,4-dione (4).

To a stirred solution of **7** (1.03 g, 1.50 mmol) in chloroform (20 mL) was added slowly NBS (0.29 g, 1.65 mmol) at 0 °C. The mixture was allowed to warm to room temperature and stirred overnight. The reaction mixture was poured into water and then extracted with chloroform. The combined organic layers were washed with water and dried over anhydrous Na_2SO_4. After filtration and evaporation, the product was purified by silica gel column chromatography (eluent: chloroform), recrystallized from chloroform/methanol, and dried under vacuum to afford **4** as a dark purple solid (yield = 973 mg, 85%). ^{1}H NMR (500 MHz, $CDCl_3$): δ 9.00 (d, *J* = 4.0 Hz, 1H), 8.61 (d, *J* = 4.0 Hz, 1H), 7.58 (d, *J* = 8.5 Hz, 2H), 7.44 (d, *J* = 4.5 Hz, 1H), 7.25 (m, 3H), 4.09-3.99 (m, 4H), 2.66 (t, *J* = 8.0 Hz, 2H), 1.94-1.89 (m, 2H), 1.67-1.61 (m, 2H), 1.41-1.29 (m, 22H), 0.93-0.89 (m, 15H). MS (MALDI-TOF) *m*/*z*: $[M]^+$ calcd 762.29; found 762.78.

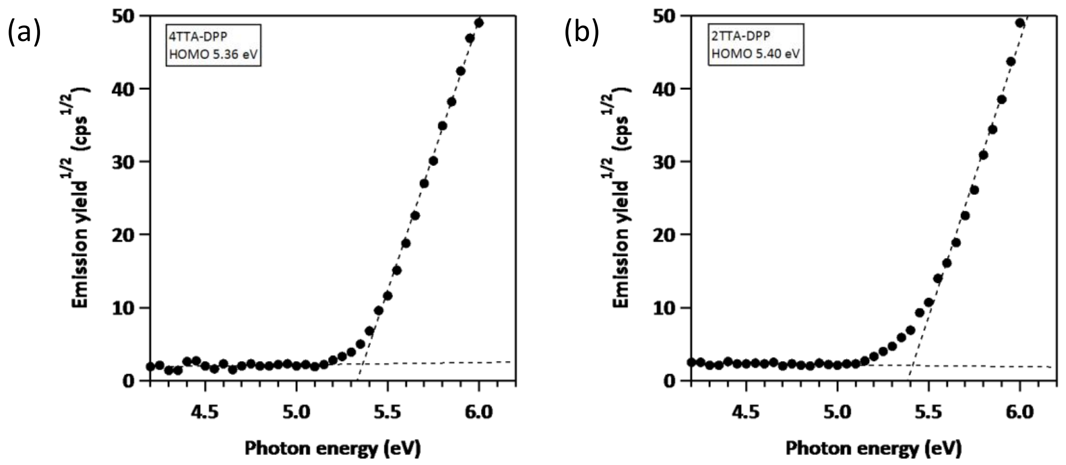

Figure S1: Photoelectron yield spectra of (a) TTA-DPP4 and (b) TTA-DPP2 thin films.

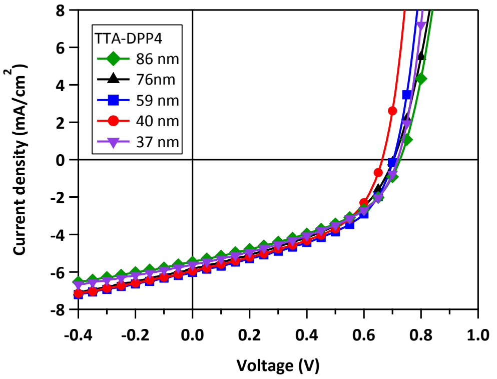

Donor	Thickness [nm]	J_{SC} [mA/cm^2]	V_{OC} [V]	FF [%]	PCE [%]
TTA-DPP4	86 ± 1	5.47	0.72	43.2	1.72
TTA-DPP4	76 ± 3	5.84	0.70	42.9	1.76
TTA-DPP4	59 ± 1	6.02	0.70	45.6	1.93
TTA-DPP4	40 ± 2	5.95	0.66	46.7	1.84
TTA-DPP4	37 ± 1	5.61	0.72	43.4	1.75

Figure S2: *J*–*V* characteristics and photovoltaic parameters of BHJ-OSCs based on TTA-DPP4:$PC_{71}BM$ (1:1.5, w/w) with different active layer thicknesses, measured under simulated AM 1.5G, 100 mW cm^{-1} illumination.

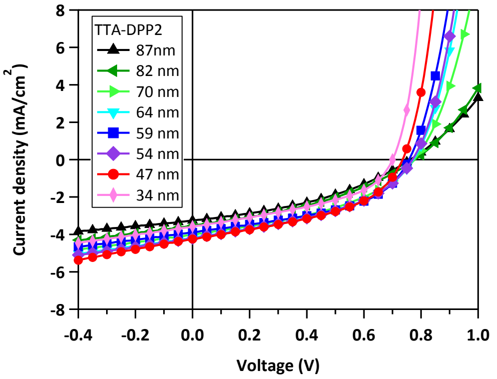

Donor	Thickness [nm]	J_{SC} [mA/cm^2]	V_{OC} [V]	FF [%]	PCE [%]
TTA-DPP2	87 ± 1	3.26	0.76	44.9	0.95
TTA-DPP2	82 ± 1	3.54	0.78	36.9	1.02
TTA-DPP2	70 ± 1	4.01	0.77	41.4	1.29
TTA-DPP2	64 ± 1	4.18	0.77	42.5	1.37
TTA-DPP2	59 ± 1	3.89	0.76	46.4	1.37
TTA-DPP2	54 ± 1	4.22	0.77	42.4	1.38
TTA-DPP2	47 ± 2	4.25	0.74	44.7	1.40
TTA-DPP2	34 ± 2	3.58	0.70	43.0	1.08

Figure S3: *J*–*V* characteristics and photovoltaic parameters of BHJ-OSCs based on TTA-DPP2:$PC_{71}BM$ (1:1.5, w/w) with different active layer thicknesses, measured under simulated AM 1.5G, 100 mW cm^{-1} illumination.

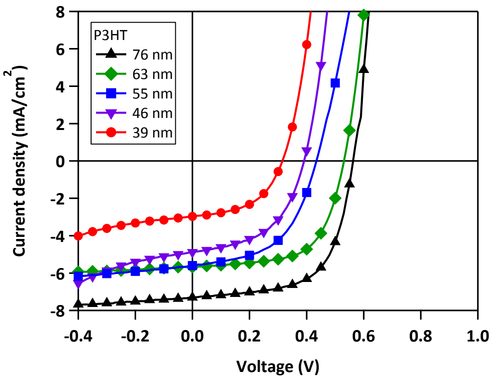

Donor	Thickness [nm]	J_{SC} [mA/cm^2]	V_{OC} [V]	FF [%]	PCE [%]
P3HT	76 ± 1	7.32	0.56	62.8	2.58
P3HT	63 ± 1	5.68	0.53	62.6	1.89
P3HT	55 ± 1	5.60	0.43	52.4	1.28
P3HT	46 ± 2	4.88	0.39	50.8	0.97
P3HT	39 ± 1	2.97	0.32	50.0	0.47

Figure S4: *J–V* characteristics and photovoltaic parameters of BHJ-OSCs based on P3HT:PC$_{61}$BM (5:3.5, w/w) with different active layer thicknesses, measured under simulated AM 1.5G, 100 mW cm^{-1} illumination.

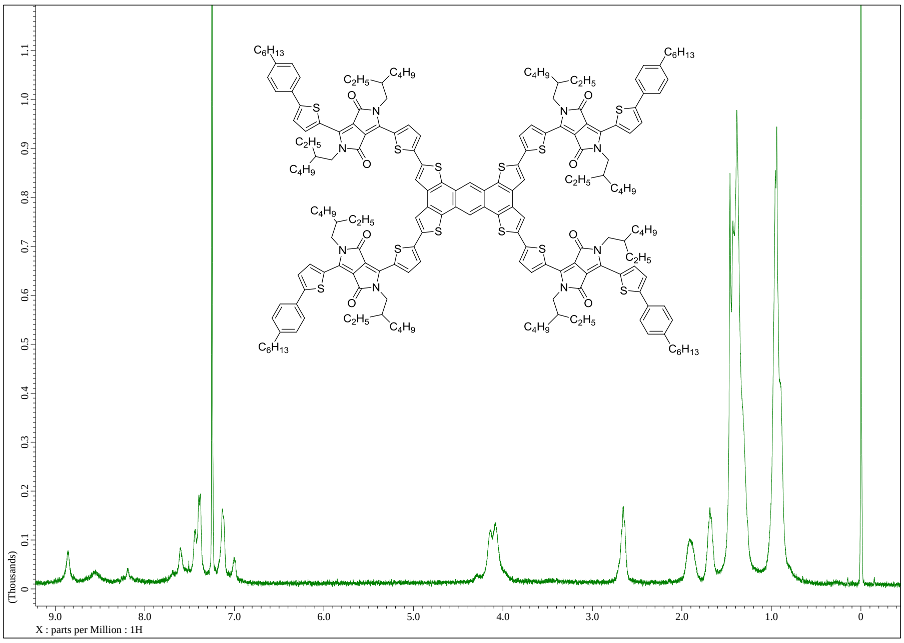

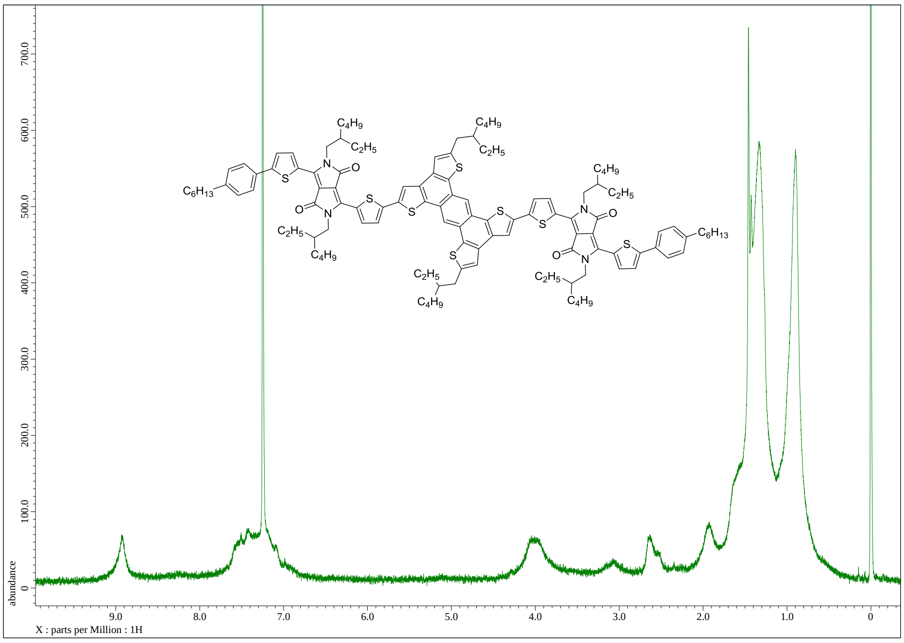

Figure S5: ^{1}H NMR spectra of (top) TTA-DPP4 and (bottom) TTA-DPP2 in $CDCl_3$, measured at 50 °C.
